# Polymer Composites Based on Polycarbonate/Acrylonitrile-Butadiene-Styrene Used in Rapid Prototyping Technology

**DOI:** 10.3390/polym15061565

**Published:** 2023-03-21

**Authors:** Katarzyna Bulanda, Mariusz Oleksy, Rafał Oliwa

**Affiliations:** Department of Polymer Composites, Faculty of Chemistry, Rzeszow University of Technology, Al. Powstańców Warszawy 6, 35-959 Rzeszów, Poland

**Keywords:** MEM, blends, hybrid materials, additive manufacturing, design

## Abstract

As part of this work, polymer composites based on polycarbonate/acrylonitrile-butadiene-styrene (PC/ABS) were obtained and used in 3D printing technology, particularly Melted Extrusion Modeling (MEM) technology. The influence of selected fillers on the properties of the obtained composites was investigated. For this purpose, modified fillers such as silica modified with alumina, bentonite modified with a quaternary ammonium salt, and hybrid lignin/silicon dioxide filler were introduced into the PC/ABS matrix. In the first part of this work, polymer blends and their composites containing 1.5–3 wt. of the filler were used to obtain the filament using the proprietary technological line. Moldings for testing the performance properties were obtained using additive manufacturing techniques and injection molding. In the subsequent part of this work, rheological properties (mass flow rate (MFR) and viscosity curves) and mechanical properties (Rockwell hardness and static tensile strength with Young’s modulus) were examined. The structures of the obtained composites were also determined by scanning electron microscopy (SEM/EDS). The obtained results confirmed the results obtained from a wide-angle X-ray scattering analysis (WAXS). In turn, the physicochemical properties were characterized on the basis of the results of tests using thermogravimetric analysis (TGA) and differential scanning calorimetry (DSC). Based on the obtained results, it was found that the introduced modified additives had a significant impact on the processing and functional properties of the tested composites.

## 1. Introduction

Additive manufacturing (AM) is still new in engineering and is favored primarily due to its ease of use and highly flexible manufacturing capabilities. Often, additive manufacturing is used to reduce production costs and shorten production times for complex prototype designs that would otherwise require expensive molds and long production times, such as in injection molding [1,2]. In response to industry demand, various additive manufacturing technologies have been developed, most of which are based on the deposition of materials in layers, such as in Melted Extrusion Modeling (MEM) technology. The technique involves melting the material, which is fed in the form of a filament, and extruding it at certain intervals and in certain places (print path), where it cools and solidifies. The process of the deposition of the material through the nozzle and the extrusion head is performed layer by layer; therefore, it is the successive overlapping of each layer that finally shapes the material into the final object [3,4,5,6]. The main differences between AM techniques include the raw material used, the states of the material and adhesion between material particles [3,4,5,6]. Despite the advances in additive manufacturing, especially in the last decade, and its adoption, primarily in engineering but also in design and education, the process is far from perfect, and there are still some challenges [4]. In order to fully realize the potential of AM technology, including MEM, the properties of the material used must be properly characterized. It is possible that this will allow the adoption of additive manufacturing in industries to produce components used in final products, including structural ones.

The most commonly used base material in composites is acrylonitrile-butadiene-styrene (ABS) [7,8]. Typically, ABS content is used to obtain strong mechanical properties, good functionality and formability [9,10,11]. On the other hand, its use is limited compared to complementary polymers (such as polycarbonate, PC) due to its lower temperature resistance and strength properties. PC is a thermoplastic polymer with properties such as creep resistance, good dimensional stability, thermal resistance and impact resistance [12,13]. ABS reinforced with PC increases the properties of the ABS material and ultimately creates a material with better mechanical properties [12,13]. Polycarbonate and acrylonitrile-butadiene-styrene blends have found many applications due to their properties as a composite material, such as high heat and impact resistance and good processability. The mechanical behavior of ABS has been improved by polycarbonate reinforcement and has enabled the development of components with excellent mechanical properties [13,14,15]. Currently, research related to the 3D printing of PC/ABS material has been conducted in many fields due to its wide application, but the PC/ABS blend lacks the quality required for manufactured parts and dynamic mechanical properties.

In the literature, there are few works on the modification of the performance properties of the PC/ABS polymer blend. Among other things, scientists have studied the effects of the content and type of filler on the mechanical, morphological and rheological properties of the polycarbonate/acrylonitrile-butadiene-styrene material by introducing glass fiber and talc into the polymer matrix [16]. The introduction of glass fiber as a filler for PC/ABS improved the tensile and flexural strength, but worsened the impact strength, while the addition of talc improved the tensile strength but weakened the flexural and impact strength. Hybrid composites containing both fillers showed the best mechanical properties. The addition of talc or glass fiber to the PC/ABS blend resulted in a reduction in the MFR, and the decrease was more dramatic with the addition of glass fiber compared to talc. The effects of untreated and treated mica fillers on the mechanical properties of polycarbonate/acrylonitrile-butadiene-styrene composites were also studied [17]. It was observed that the surface treatment of mica with a coupling agent in the PC/ABS composite improved the tensile stress, elongation at break, flexural strength and impact strength. Moreover, in [18], scientists attempted to investigate the effects of two flame retardants, ammonium polyphosphate (APP) and montmorillonite (MMT), on the flame retardancy, mechanical properties and physical properties of PC/ABS blends. Based on these studies, it was shown that the presence of APP and MMT in the polymer matrix significantly improved the limiting oxygen index (LOI). It has also been shown that the content of fillers in PC/ABS blends can improve thermal stability, tensile strength and elongation at break. The improvement of mechanical properties after introducing small amounts of MMT and small amounts of bentonite into the PC/ABS matrix has also been described by other scientists [19,20]. Researchers interested in electrically conductive composites [21] investigated the effects of graphene, carbon nanotubes and soot on the PC/ABS matrix. They observed an improvement in the dielectric properties of the composites in terms of the loss factor with increasing temperature and filler concentration, as well as a beneficial effect on the improvement of the tensile strength and a decrease in the impact strength. The presented results were also confirmed by other researchers [22,23,24,25]. Graphene nanoplates can also be successfully introduced into PC/ABS to improve the thermal conductivity of composites [26,27].

In conclusion, there are few publications on modifying the properties of the PC/ABS mixture. No works were found in which the polymer blend was modified for the use of the material in 3D printing in MEM technology. Therefore, as part of this work, polymer composites based on polycarbonate/acrylonitrile-butadiene-styrene with the addition of selected modified nanofillers and fillers were developed and obtained, and the performance properties of the obtained polymer composites were precisely characterized. The fillers were selected to improve the processing properties (fluidity) of PC/ABS, as well as its thermal stability, while maintaining good physical and chemical properties. This article is a continuation of earlier works [28,29,30].

## 2. Materials and Methods

### 2.1. Materials

Commercial polycarbonate/acrylonitrile-butadiene-styrene blend (Natural, Filamentum Manufacturing Czech s.r.o., Hulin, Czech Republic) was used as the polymer matrix (PC/ABS).

PC/ABS was filled with:

- S: silica containing alumina (Aerosil MOX 170, Evonic Industries, Hanau, Germany);
- B: bentonite (technical product “Specjal”, Zębiec SA Zakłady Górniczo-Metalowe, Zębiec, Poland) modified with quaternary ammonium salt (BARQUAT^®^ DM80, Lonza, Switzerland);
- L: lignin hybrid filler (Sigma-Aldrich, Burlington, MA, USA)/silicon dioxide (Syloid 244, WR Grace & Co., Baltimore, MD, USA);
- E926: compatibilizer—chemically modified polyethylene grafted with maleic anhydride (Fusabond E926, DuPont, Wilmington, DE, USA).

Modified fillers were introduced into PC/ABS to improve the thermal stability and fluidity of the composites (Table 1).

### 2.2. Preparation of Composites and Samples

The dried components of the composite (PC/ABS: 90 °C, 4 h; fillers S, B, L: 200 °C, 24 h) were homogenized on a Coperion twin-screw extruder with the following parameters: temperature from 230 °C to 260 °C, extrusion capacity of 4 kg/h and screw speed of 400 rpm. The granules thus obtained were dried under vacuum (90 °C; 4 h). From the thus-obtained and dried composites, filaments were obtained on a specially designed, together with Gamart SA (Jasło, Poland), proprietary technological line dedicated to the production of filaments. The process was carried out in an extrusion temperature range from 235 °C to 260 °C, obtaining fibers with a diameter of approx. 1.75 ± 0.05 mm. The proprietary technological line has already been presented in other works [28,29,30].

**Figure 1:**
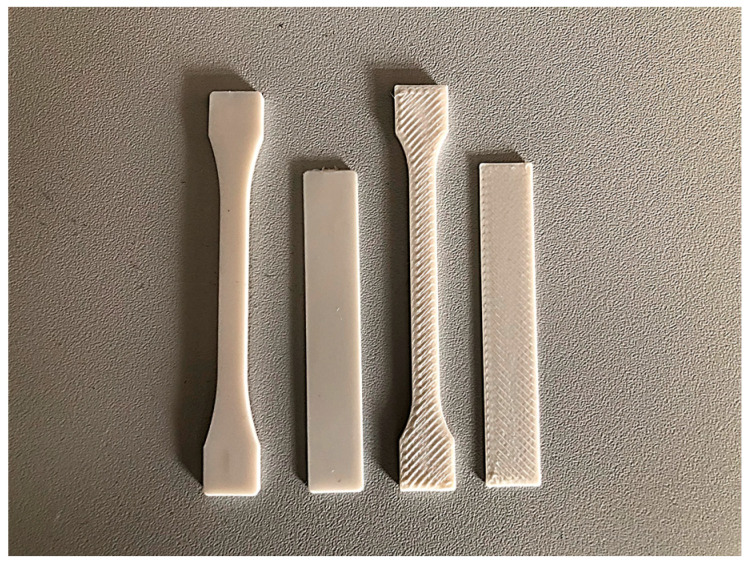
Samples obtained by injection molding or 3D printing.

The obtained composites were used to make samples (bears and paddles) necessary for the research. The shapes of the obtained samples are shown in Figure 1.

The samples were obtained with two techniques, 3D printing using the UP BOX (UP BOX, TierTime, Beijing, China) printer, which works with MEM technology, and injection molding using the Haake MiniJet II injection molding machine (Waltham, MA, USA).

**Figure 2:**
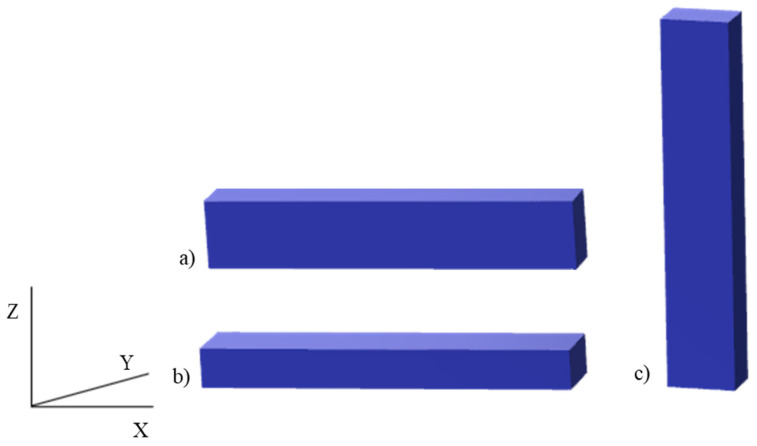
Print orientation: (**a**) edge (Y), (**b**) horizontal (X), (**c**) vertical (Z). Own elaboration based on [19].

**Figure 3:**
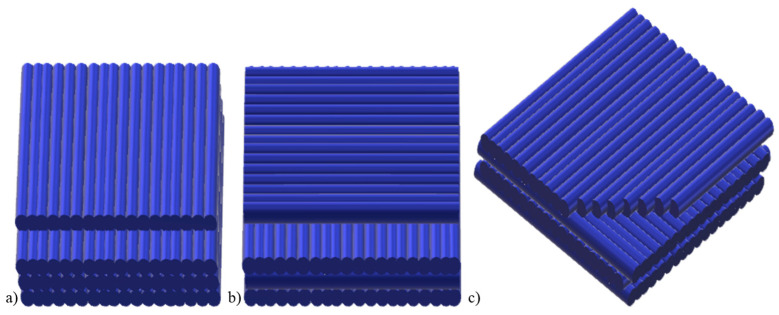
Raster orientation: (**a**) 0°, (**b**) 90°, (**c**) −45°/−45°. Own elaboration based on [31].

The samples obtained by additive manufacturing were placed on the printer’s worktable in a horizontal orientation (Figure 2b) and a raster orientation of +/−45° (Figure 3c).

The parameters of the 3D printing and injection molding processes are summarized in Table 2.

### 2.3. Method Characterization

The melt flow rate, MFR, was determined using a plastometer (DYNISCO 4781, Kayeness INC., Honey Brook, PA, USA). For this purpose, samples weighing about 4 g were introduced into the apparatus heated to 260 °C, and then a preload of 1.1 kg was applied for 240 s. After this time, the load was changed to the appropriate 2.16 kg, and the measurements started. The sample squeezed out of the nozzle was cut off after a given time and then weighed. For each series, three measurements were made in accordance with the ISO 1133 standard.

Shear viscosity was measured using a capillary rheometer (Smart RHEO, Instron Ceast, Norwood, MA, USA). Samples weighing about 10 g were introduced into the apparatus heated up to 260 °C, where they were thermostated for 300 s under the preload of the piston. The measurement was carried out for a shear rate range from 100 1/s to 3000 1/s. A capillary with a length of 40 mm and a width of 1.16 mm was used for the measurement. The test was carried out in accordance with the 11443:2005 standard.

Rockwell hardness was measured using a hardness tester (Zwick/Roell, Zwick GmbH & Co., Ulm, Germany) at ambient temperature. The sample was placed in the apparatus, the specified load was applied (the load at which the indenter collapsed to a thickness of 0.15–0.35 mm), and a 30 s measurement was started. For each lot, ten determinations were made in accordance with the ISO 6508 standard.

The determination of the strength properties during the static tensile test was carried out on a tensile testing machine (INSTRON 5967, Grove City, PA, USA) at ambient temperature. The paddle-shaped samples were placed in machine holders; Young’s modulus was measured at a tensile rate of 5 mm/min (to achieve 1% tensile strain), and the speed was increased to 50 mm/min. For each series, five measurements were made in accordance with the ISO 527 standard.

Microstructural observations were carried out using a scanning electron microscope (Hitachi TM3000, Red Star Vietnam Co., Ltd., Hanoi, Vietnam) with an energy-dispersive spectroscopy (EDS) microanalysis apparatus. Brittle fractures obtained by impact cracking of the sample after it was cooled in liquid nitrogen were used for the observations. Before the observations, samples of the brittle fractures of the polymers and composites were sputtered with a layer of gold with palladium. The observations were carried out at a voltage of 5 keV.

Wide-angle X-ray diffraction (WAXS) measurements were performed using a diffractometer (NanoStar-U, Bruker Inc., Billerica, MA, USA) with a two-dimensional detector in transmission geometry. X-ray radiation with a wavelength of 1.54 Å was produced by irradiating a copper tube powered by 600 µA at 50 kV. Measurements were made at room temperature (about 22 °C). The scattering angle range was from 0° to 28°. Fillers and polymer composites with their addition were tested.

Thermogravimetric analysis was performed with a TGA/DSC 1 (Mettler Toledo DSC 1 Star^®^ System, METTLER Toledo, Schwerzenbach, Switzerland) under a nitrogen atmosphere. In the first apparatus, 5 mg samples were heated on platinum plates from 25 °C to 600 °C at a rate of 10 °C/min.

Differential scanning calorimetry (Mettler Toledo DSC 1 Star^®^ System, METTLER Toledo, Schwerzenbach, Switzerland) was also performed. Measurements were made in a helium atmosphere in hermetic aluminum crucibles. About 6 mg samples were heated from −90 °C to 300 °C at 10 °C/min, cooled to −90 °C at 10 °C/min, and then reheated to 300 °C at 10 °C/min.

## 3. Results

In order to investigate the influence of the fillers used (1.5% and 3% of the additive) on the fluidity of the composites obtained on the basis of PC/ABS, the melt flow rate was determined, and the obtained results are summarized in Table 3.

The literature confirms that the mass flow rates of composites with the addition of mineral fillers show an increasing tendency up to 3% filler content. The introduction of larger amounts of these fillers into the polymer matrix may cause a decrease in the fluidity of the material [32,33].

Based on the obtained test results, it was found that composites containing modified fillers (3%S, 3%B, 3%L, and 1.5%L/1.5%B) are characterized by an improved value of the melt flow rate, which coincides with results described in the literature [34,35]. The introduction of only 3%S to the polymer matrix resulted in a slight increase of 33.2% compared to PC/ABS, while subsequent composites achieved results of over 100% higher. Furthermore, using the hybrid filler 1.5%L/1.5%B, the best results among the tested materials were obtained, reaching even 163.9% higher compared to the base polymer.

**Figure 4:**
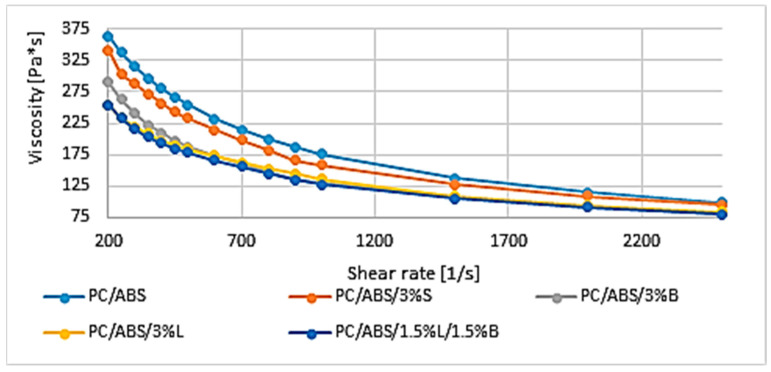
Viscosity curves of PC/ABS and composites based on PC/ABS.

In the next stage of the research, the viscosity-versus-shear-rate curves were determined for the PC/ABS blend and the composites based on it (Figure 4).

It can be noticed that the viscosity of the tested systems decreases with increasing shear rate. All composites are characterized by lower viscosity compared to the polycarbonate/acrylonitrile-butadiene-styrene matrix, and the lowest value of the tested parameter was obtained for PC/ABS/1.5%L/1.5%B, from a viscosity of 254.7 Pa·s at a shear rate 200 s^−1^ to 79.9 Pa·s at 2500 s^−1^. Slightly better results were obtained for composites filled with modified lignin (3%L) or modified bentonite (3%B). The obtained flow curves agree with the previously discussed MFR results (Table 3). For PC/ABS/3%B, PC/ABS/3%L, and PC/ABS/1.5%L/1.5%B, a significant difference in viscosity (of about 100 Pa·s) was obtained in the initial course of the curves at small shear rates of 200–700 s^−1^. However, in the final stage, at improved shear rate values of 2000–2500 s^−1^, the differences in the flow resistance of the composites decrease, and thus, the obtained curves for individual materials are characterized by a similar course.

Three-dimensional printing provides many advantages in the process, such as reduced costs and shortened production times for geometrically complex prototype designs, which, in traditional methods, such as injection molding, would require the use of expensive molds and long production times. However, the details made by rapid prototyping technologies still do not match the properties of injection-molded elements. This observation is widely described in the literature [36,37,38] and results from the greater homogeneity of the samples obtained by injection into the mold [39]. However, by developing and obtaining new materials, it is possible to obtain sufficient characteristics of details for a dedicated application. Researchers often compare the two technologies, especially when discussing mechanical properties, so that readers can determine for themselves whether the material of interest has the appropriate properties or whether traditional manufacturing methods will be required. For this reason, the results obtained with these two techniques are presented separately.

**Figure 5:**
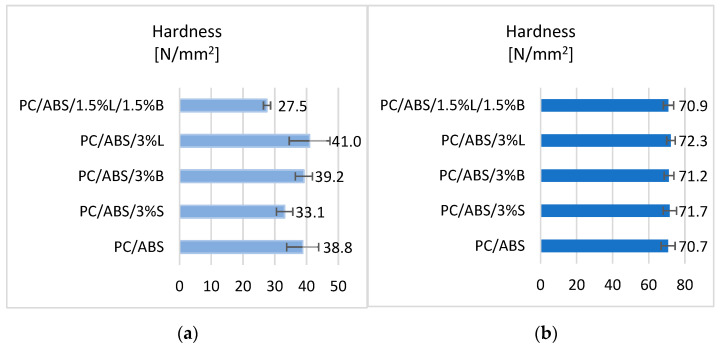
Hardness test results of (**a**) samples obtained by 3D printing and (**b**) samples obtained by injection molding.

The Rockwell hardness results obtained for PC/ABS and PC/ABS-based composites are presented in the form of graphs (Figure 5).

Analyzing the obtained results, it can be seen that the introduction of fillers in order to modify the properties of the PC/ABS polymer blend affected the obtained hardness values. In the case of hardness tests for fittings obtained with the rapid prototyping technology (Figure 5a), it was noticed that the PC/ABS/3%L composite obtained a 5.6% better result compared to PC/ABS. The lowest Rockwell hardness was obtained for the composite containing the hybrid filler 1.5%L/1.5%B (27.5 N/mm^2^). In the case of the results obtained for moldings produced by injection molding (Figure 5b), it was observed that all obtained composites are characterized by higher hardness compared to the unfilled polycarbonate/acrylonitrile-butadiene-styrene matrix.

**Figure 6:**
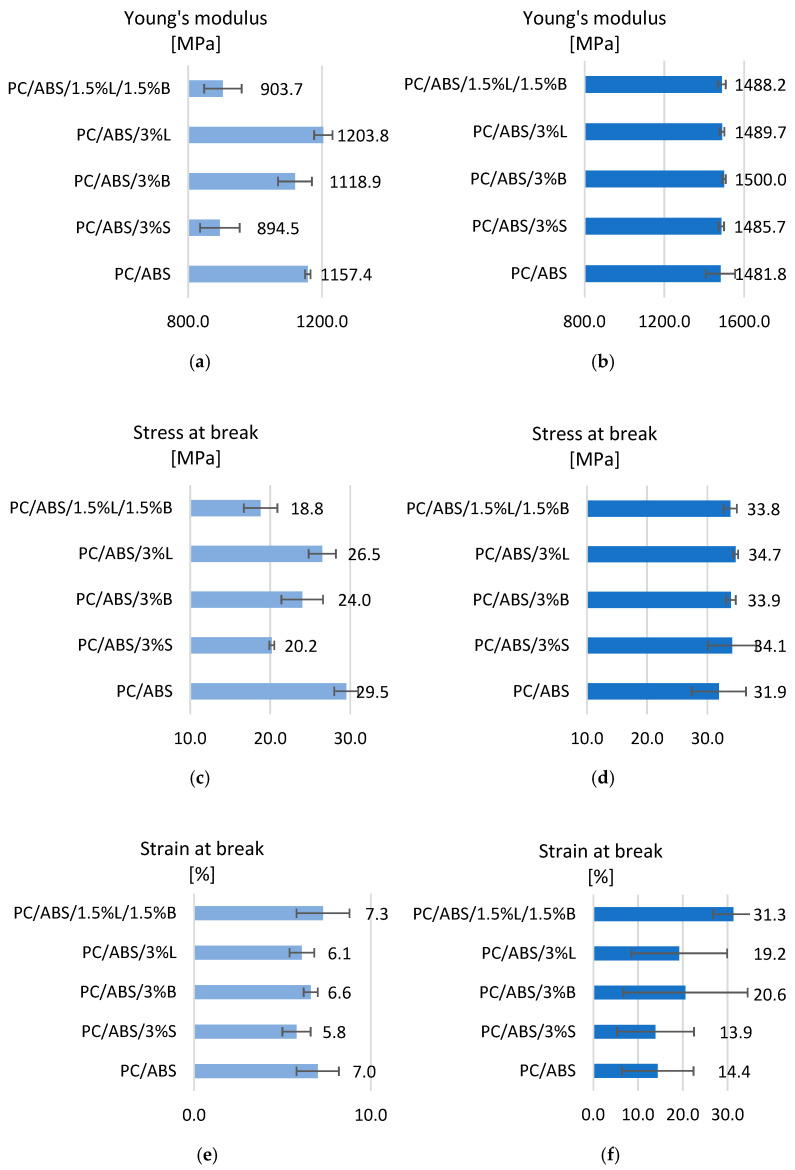
Results of static tensile strength tests: Young’s modulus of (**a**) samples obtained by 3D printing and (**b**) samples obtained by injection molding; stress at break of (**c**) samples obtained by 3D printing and (**d**) samples obtained by injection molding; strain at break of (**e**) samples obtained by 3D printing and (**f**) samples obtained by injection molding.

Figure 6 presents the results of mechanical tests during the static tensile test for the polycarbonate/acrylonitrile-butadiene-styrene blend and PC/ABS composites. Analyzing the results obtained for the moldings produced with MEM technologies, it was found that the introduced fillers affect the Young’s modulus obtained during the static tensile test (Figure 6a). Only in the case of the PC/ABS/3%L composite was a better result obtained compared to the unfilled polymer blend, as the value of the tested feature improved by 5.8%. Other composites are characterized by a decrease in the modulus by as much as 21.4%. The lowest Young’s modulus values were obtained for PC/ABS composites with the addition of 3%S and 1.5%L/1.5%B. The obtained results are consistent with the hardness of the composites, because PC/ABS/3%L (41.0 N/mm^2^) is characterized by the highest values of the tested parameter, while those of PC/ABS/3%S (33.1 N/mm^2^) and PC/ABS /1.5%L/1.5%B (27.5 N/mm^2^) are the lowest. Moreover, when analyzing the Young’s modulus results obtained for moldings made by injection molding, it was observed that the introduced fillers did not significantly affect the stiffness of the blend (Figure 6b). The most rigid material turned out to be PC/ABS/3%B, for which a modulus value of 1500.0 MPa was obtained.

The analysis of tensile-stress-at-break tests shows that the modified fillers had a negative impact on the test results, because a better result was obtained for the unmodified PC/ABS blend (29.5 MPa). The composites showed lower stress, ranging from 18.8 MPa to 26.5 MPa (Figure 6c). The obtained polymeric materials are also characterized by a lower breaking deformation, ranging from 5.7% to 17.9%, compared to the matrix. Only when the 1.5%L/1.5%B filler was introduced into the matrix was a deformation higher than that of PC/ABS obtained, equal to 7.3% (Figure 6e). As part of these mechanical tests, the tensile stress and strain at break were also determined for moldings obtained by injection (Figure 6d,f). It was observed that the introduction of modified fillers positively influenced the results of tensile stress; an improvement in the range of 6.1% to 9.0% was noted compared to the PC/ABS polymer blend. An increase in the breaking strain was also obtained for these tested composites. It turns out that the exception is the PC/ABS/3%S composite, whose deformation value is 13.9%.

**Figure 7:**
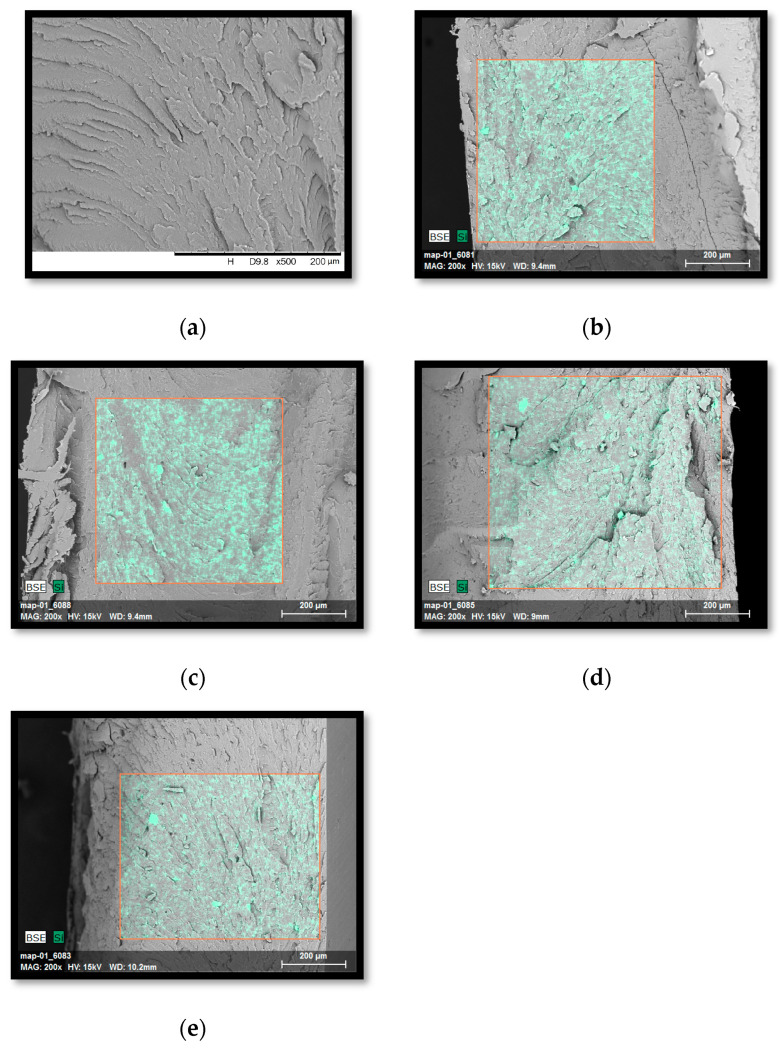
SEM micrographs with the EDS adapter of the PC/ABS blend and composites based on it: (**a**) PC/ABS, (**b**) PC/ABS/3%S, (**c**) PC/ABS/3%B, (**d**) PC/ABS/3%L, (**e**) PC/ABS/1.5%L/1.5%B. The red contour marks the area subjected to EDS analysis, which was performed in order to observe the degree of dispersion of the filler and the distribution of the silicon element.

On the basis of SEM micrographs of brittle fractures of the tested composites, it was observed that the unfilled polymer blend (Figure 7a) is characterized by a fracture containing numerous grooves.

The introduction of modified S, B, L, and L/B fillers (Figure 7b–e) resulted in more ragged plaques. It is difficult to distinguish between the occurring phases (polymer and additive), and therefore, the EDS system was used, which allows the determination of the elemental composition of the top layer of the composite. Since the fillers used contain silicon in their chemical structure, the decomposition of this element was observed in the SEM/EDS images. As already mentioned, the selected test area was marked in red, on the basis of which a good dispersion of the introduced modified fillers was found. No clusters or agglomerates of fillers were observed, which proves that the conditions of their homogenization in the PC/ABS matrix were well selected.

**Figure 8:**
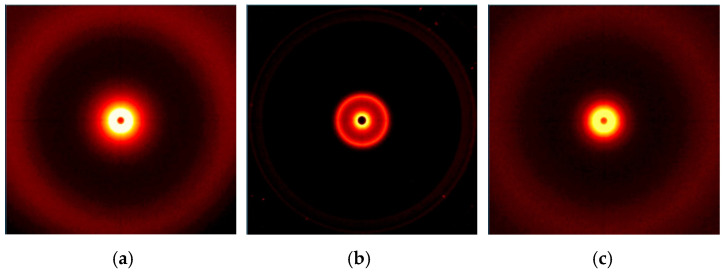
Results of 2D WAXS imaging of the fillers used: (**a**) S, (**b**) B, (**c**) L.

**Figure 9:**
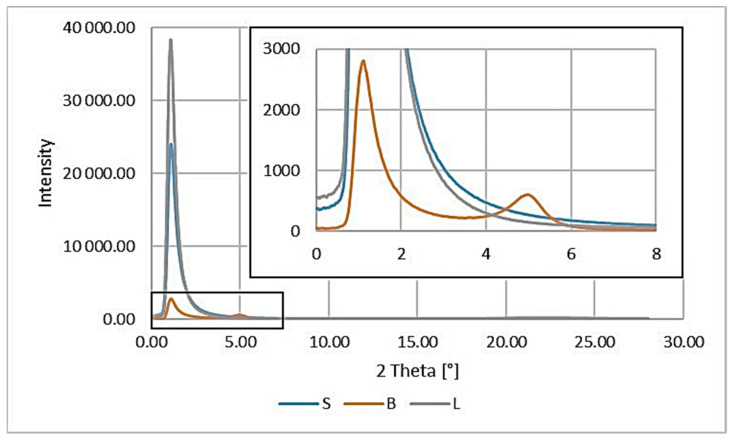
Results of the WAXS study of the fillers used.

Wide-angle 2D X-ray diffraction patterns of the fillers are shown in Figure 8, and graphs of radiation intensity as a function of the scattering angle are shown in Figure 9.

The range of scattering angles for the tested materials was from 0° to 28°, in which peaks were observed on the curves only for modified bentonite B (Figure 9). In the 2D-WAXS images (Figure 8a,c) the presence of modified silica (S) and lignin (L) in this range was also noted, but the obtained intensity was too low to record their scattering angles.

The distance between successive planes of the filler (d_hkl_) was calculated from the Bragg formula:

(1) $$d_{\mathrm{hkl}}=\frac{n\lambda }{2\sin\theta }$$

where *n* is the degree of diffraction (*n* = 1, 2...), λ is the wavelength of radiation used and 2θ is the angle at which the diffractive peak occurs, as read from the WAXS graph.

The particle size in the Scherrer formula was also determined:

(2) $$D_{\mathrm{hkl}}=\frac{K\lambda }{\mathrm{bcos}\theta }$$

where D_hkl_ is the reflex width depending on the size of crystallites, K is Scherrer’s constant, K = 1, λ is the wavelength of radiation used and b is the half-width of the diffraction peak for the plane (hkl).

Analyzing the results obtained for the fillers (Figure 9), we can see a peak for B at a value of 5.0°, which can be attributed to the diffraction reflection from the bentonite (001) sheets [40]. Unfortunately, it was not possible to determine the characteristic peaks for the other modified fillers (S and L) due to the determination conditions used and the technical capabilities of the apparatus (range of scattering angles from 0° to 28°).

The distances between successive packets of the filler plates and the size of their particles were calculated: for B d_khl_, the value is 18.2 Å, while for D_khl_, it is 110.8 Å.

**Figure 10:**
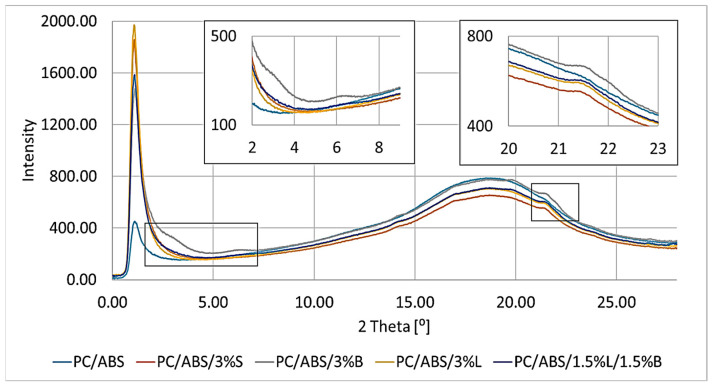
Results of the WAXS study of the composites.

When analyzing the presented results of the WAXS study for the PC/ABS blend and composites based on it, it was noticed that the introduced fillers were well dispersed in the polymer matrix (Figure 10), which confirms the previously presented SEM/EDS results.

On the WAXS curves of the obtained composites, a wide peak with a 2θ value of about 18° was observed, which is a characteristic result for the arrangement of the PC/ABS polymer [41]. In addition, the PC/ABS/3%B composite is characterized by small 2θ peaks of approximately 2.9° and 6.2°, while the analysis of the results obtained for fillers revealed that the filler B has a band at 5°. It can therefore be concluded that only the bentonite layers in the composite were separated, but not fully dispersed. The distances between successive packets of filler plates and their particle sizes were calculated for a 2θ value equal to 2.9°: for B d_khl_, the value is 31.1 Å, and for D_khl_, it is 64.8 Å. Comparing the obtained d_khl_ and D_khl_ results with those calculated for the filler, an increase in the distance between the bentonite plate packages of 12.9 Å and a decrease in particle size of 46.0 Å were observed. For the obtained composites, small peaks were also obtained at 21.5°, which was not observed in the case of the unfilled polymer matrix. When observing this phenomenon, it was found that the obtained peaks came from the introduced compatibilizer, which was polyethylene grafted with maleic anhydride. The obtained band corresponds to the literature values for PE (2θ = 21.7°) [41].

**Figure 11:**
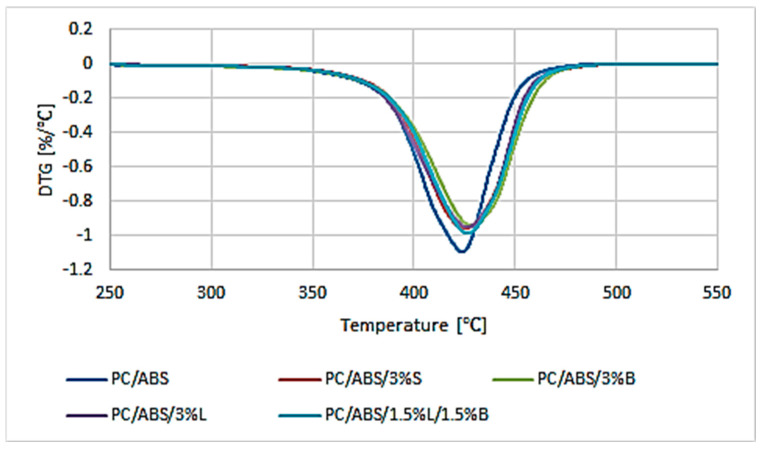
Results of DTG analysis (mass change derivative curve) of PC/ABS and composites based on PC/ABS.

The results of testing the thermostability properties of the composites are presented in Table 4 and Figure 11. The temperatures of 2% mass loss (T_2%_) and 5% mass loss (T_5%_), which can be taken as the loss of volatile substances and the beginning of the degradation process, respectively, were determined from the TGA curve. The maximum temperature of the T1 degradation stage and the rate of degradation at the maximum temperature (ΔV_1_) were determined from the mass change derivative curve. The residue from the initial mass of the sample obtained at 600 °C was also determined (R_600_).

When analyzing the results obtained for the polymer blends and composites based on it, the one-stage thermal decomposition of the tested materials was observed (Figure 11). The stage of the composite degradation process is characterized by a mild peak, and only in the case of PC/ABS was a slightly intense peak obtained, appearing in the temperature range of 380–485 °C The unmodified polymer blend is characterized by good thermal stability, because for PC/ABS, the temperature of the loss of volatile substances was read at 344.3 °C, and the start of the degradation process occurred at 383.4 °C (Table 4). It should be noted that, in the literature, one can find works describing similar thermal stability of the PC/ABS blend [42,43]. The introduced modified fillers S, B, and L and the hybrid L/B system improved the thermal stability of the materials. This indicates a positive interfacial interaction between the filler and the PC/ABS matrix. This phenomenon proves the appropriate dispersion of additives in the polymer matrix, which is also confirmed by SEM studies. The best result of thermal stability tests was obtained for the PC/ABS/3%S composite, for which the temperature at the beginning of the degradation process is 390.6 °C, which is an increase of 7.2 °C compared to PC/ABS. This may be related to the relatively large specific surface of the silica filler. The presence of 3%S in the polymer matrix leads to the formation of an interlayer zone on the surface of the filler and thus to the immobilization of polymer chains.

**Figure 12:**
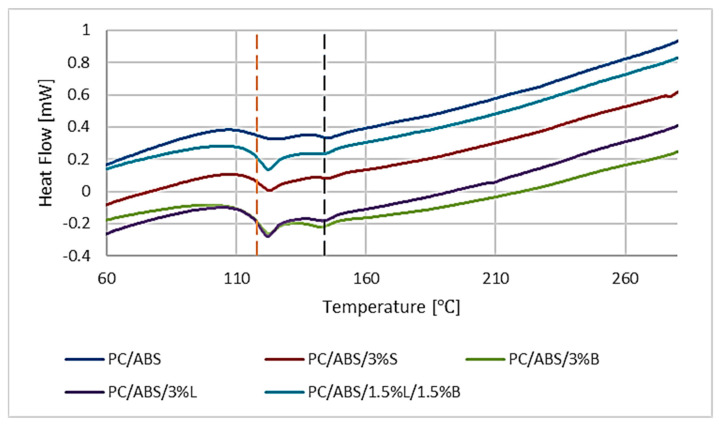
Results of the differential scanning calorimetry (DSC) analysis of PC/ABS and PC/ABS composites.

Figure 12 shows the DSC thermograms of the second heating of the PC/ABS polymer blend and the composites based on it. The inflection observed at about 145 °C is related to the PC glass transition. ABS is a copolymer with two glass transition temperatures, one for the polybutadiene phase and one for the styrene-acrylonitrile phase. Due to the test conditions used, it was not possible to determine T_g_ for polybutadiene, which is about −80 °C. On the other hand, visible bends at a temperature of about 118 °C may be associated with the T_g_ of the styrene-acrylonitrile phase [44]. No significant changes in glass transition temperatures were observed for any of the tested composites compared to the unfilled polymer blend.

## 4. Conclusions

The main purpose of this work was to develop and then obtain innovative polymer composites based on PC/ABS with the addition of modified nanofillers for MEM technology. Fillers that are very well described in the literature were selected as supplements to the matrix. From the composites prepared in this way, filaments for 3D printing were obtained using a technological line specially developed and obtained for this purpose.

The effect of the addition of three different modified fillers, namely, silica modified with alumina, bentonite modified with a quaternary ammonium salt, and a lignin/silicon dioxide hybrid filler, on the properties of the obtained polycarbonate/acrylonitrile-butadiene-styrene composites was investigated.

The addition of modified fillers to the polymer matrix improved the fluidity of the material. The melt flow rate improved from 33.2% for PC/ABS/3%S by as much as 163.9% for PC/ABS/1.5%L/1.5%B compared to unfilled PC/ABS. As expected, a decrease in the viscosity of the obtained polymer composites was also observed after adding additives to PC/ABS. The lowest value of the tested parameter was obtained for PC/ABS/1.5%L/1.5%B, where the viscosity was 254.7 Pa·s at a shear rate of 200 s^−1^ and 79.9 Pa·s at 2500 s^−1^. A general increase in the Rockwell hardness of the obtained composites was observed for both 3D-printed and injection-molded moldings. Only in the case of samples obtained by additive manufacturing from PC/ABS/3%S (33.1 N/mm^2^) and from PC/ABS/1.5%L/1.5%B (27.5 N/mm^2^) was a lower hardness obtained compared to the polymer matrix. The introduced fillers had a negative impact on the obtained Young’s modulus determined during the static tensile test for moldings produced with MEM technologies. Only in the case of the PC/ABS/3%L composite was a better result obtained compared to the polymer blend, as the value of the tested feature improved by 5.8%. The other composites are characterized by a decrease in the modulus of up to 21.4%. In addition, when analyzing the results of the Young’s modulus obtained for moldings made by injection, it was noticed that the introduced fillers did not significantly affect the stiffness of the mixture. The most rigid material turned out to be PC/ABS/3%B, for which a modulus of 1500.0 MPa was obtained. Observations of the microstructures of composites using the SEM/EDS method confirmed the uniform distribution of fillers in the polymer matrix, and the results also confirmed the results obtained from the WAXS study. The TGA results show that all materials are characterized by single-stage thermal decomposition. The introduced modified fillers S, B, and L and the hybrid L/B system improved the thermal stability of the materials, although the polymer matrix itself is characterized by good thermal stability, because for PC/ABS, the loss of volatile substances was read at 344.3 °C, and the beginning of the degradation process was observed at 383.4 °C. The best TGA result was obtained for the PC/ABS/3%S composite, for which the temperature at the beginning of the degradation process is 390.6 °C, which means an increase of 7.2 °C in relation to PC/ABS. The DSC test showed that PC/ABS is characterized by phase changes typical for the material, and the introduced additives did not cause significant changes in the glass transition temperatures of the tested materials.

## Figures and Tables

**Table 1:** Compositional data of the composites.

Composition	PC/ABS Content (wt.%)	S Content (wt.%)	L Content (wt.%)	B Content (wt.%)	E926 Content (wt.%)
PC/ABS	100	-	-	-	-
PC/ABS/3%S	96	3	-	-	1
PC/ABS/3%B	96	-	-	3	1
PC/ABS/3%L	96	-	3	-	1
PC/ABS/1.5%L/1.5%B	96	-	1.5	1.5	1

**Table 2:** Three-dimensional printing and injection molding parameters.

3D Printing		Injection Molding	Paddles	Bars
Nozzle diameter	0.4 mm	Mold temperature	70 °C	70 °C
Layer height	0.2 mm	Injection temperature	260 °C	260 °C
Infill percentage	100%	Injection pressure	800 bar	850 bar
Infill pattern	±45°	Post pressure	700 bar	750 bar
Extrusion temperature	260 °C	Plasticizing time	120 s	120 s
Bed temperature	90 °C	Injection time	5 s	5 s
Printing speeds	70 mm/s	Post time	3 s	3 s

**Table 3:** Results of the mass flow rate of the obtained materials.

Composition	PC/ABS	PC/ABS/3%S	PC/ABS/3%B	PC/ABS/3%L	PC/ABS/ 1.5%L/1.5%B
MFR	2.2	3.0	4.7	4.9	5.6
(g/10 min)	±0.1	±0.0	±0.1	±0.1	±0.1

**Table 4:** The results of research on the properties of the thermostability of composites.

Composition	T_2%_ (°C)	T_5%_ (°C)	T_1_ (°C)	ΔV_1_ (%/°C)	R_600_ (%)
PC/ABS	344.32	383.40	431.10	1.10	3.71
PC/ABS/3%S	365.52	390.63	442.02	0.97	8.26
PC/ABS/3%B	357.44	387.64	443.38	0.94	6.94
PC/ABS/3%L	362.33	388.79	442.32	0.95	6.87
PC/ABS/ 1.5%L/1.5%B	361.18	388.80	442.52	0.98	6.87

## Data Availability

The data presented in this study are available on request from the corresponding author.
